# Articular impactions in acetabular fractures

**DOI:** 10.1007/s00402-024-05611-z

**Published:** 2024-12-12

**Authors:** Axel Gänsslen, Thomas Freude, Jan Lindahl, Dietmar Krappinger

**Affiliations:** 1 Trauma Department, Hannover Medical School, Hannover, Germany; 2 Department of Trauma and Orthopedics, Johannes Wesling Hospital, Minden, Germany; 3 University Hospital for Orthopaedics and Traumatology, Paracelsus Medical University, Salzburg, Austria; 4 Department of Orthopaedics and Traumatology, Helsinki University Hospital and University of Helsinki, Helsinki, Finland; 5 Department of Orthopaedics and Traumatology, Medical University Innsbruck, Anichstraße 35, 6020 Innsbruck, Austria

**Keywords:** Acetabular fracture, Marginal impactions, Femoral head impactions, Reduction, Clinical relevance

## Abstract

Impactions of the articular surface are relevant prognostic parameters in the treatment of acetabular fractures. Posterior marginal impactions and acetabular dome impactions may occur depending on the direction of the force vectors during trauma. Posterior marginal impactions are mainly observed in posterior fracture dislocations, while acetabular dome impactions are frequently seen in the elderly with the hip joint in extension during trauma. Femoral head impactions are also frequently associated with acetabular fractures, mainly in fracture dislocations and transverse acetabular fractures. CT scans using thin slices are mandatory in order to preoperatively identify acetabular and femoral head impactions. Intraoperatively, the reduction techniques depend on the type of marginal impaction. Posterior impactions are usually addressed via a posterior approach by applying femoral traction under direct visualization or even by performing surgical hip dislocation. Acetabular dome impactions may be reduced using the fracture lines or by creating a cortical window. Reduction is followed by filling the void with bone or bone substitutes supported by raft screws. No clear treatment recommendations for femoral head impactions are given in the literature.

## Introduction

It is well known that acetabular and accompanying femoral head impactions negatively affect the clinical outcome of acetabular fractures [1, 4, 39]. Surprisingly, there is a relative paucity of literature dealing with these impactions. Emile Letournel described acetabular impactions as “the impaction and incarceration into the underlying cancellous bone of small osteochondral fragments from the shattered margin of the acetabulum “ [26]. Such accompanying impactions were frequently observed in fracture dislocations with a rate of 16% [26]. Letournel described marginal impactions mainly in posterior fracture types, but also in an anterior wall fracture and in anterior column fractures with a gull-sign configuration [26]. In his later work, he additionally focused on thin CT slices (1 mm) “to study incarcerated fragments or impactions of the femoral head”. It was noted that “CT is the ideal means for evaluating marginal impactions of the articular surface, which are so frequent in acetabular fractures [27].”

In 2003, Anglen reported on the “sea-gull sign” as a superomedial dome impaction [1]. It was found that the presence of a gull sign resulted in loss of reduction after open reduction and internal fixation of acetabular fractures in patients over 60 years in all cases [1]. Anglen stated the following: “The displaced fragment identified by the “gull sign” is difficult to reduce and fix for two reasons. In the first place, the superoanteromedial location of the fragment and its displacement into the cancellous bone renders it difficult to access. Occasionally it can be reached through the fracture lines via an ilioinguinal exposure. Secondly, once reduced, the articular fragment has no supporting cancellous bone and easily redisplaces. It is difficult or impossible to get any bone graft or hardware applied in a manner to reliably support the fragment” [1].

**Fig. 1:**
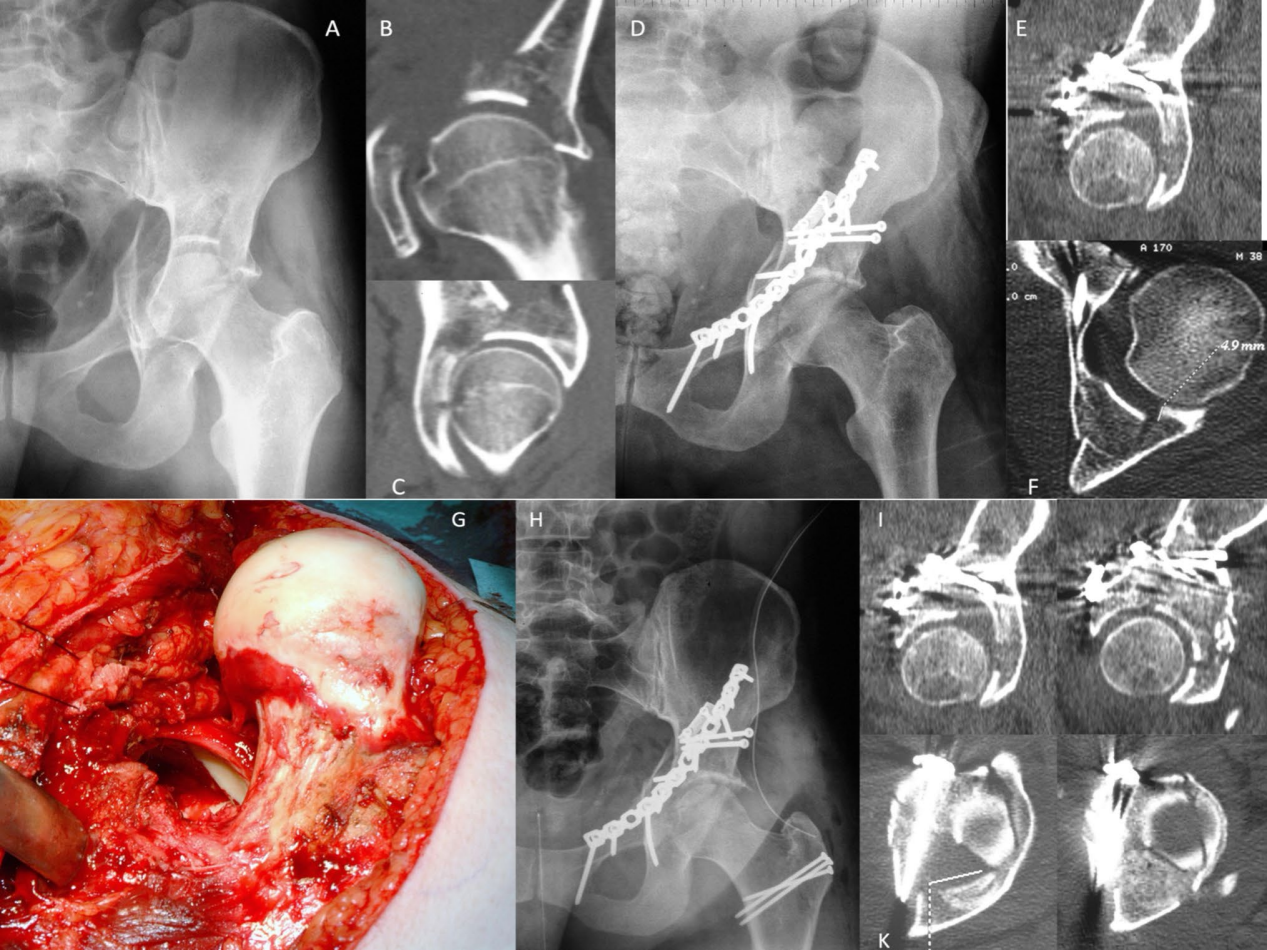
35 year old male sustaining a simple fall on the left lateral hip. Imaging shows an anterior column fracture (**A**) with marginal impactions cranially (**B**) and posteriorly, while the posterior column was intact (**C**). Open reduction and internal fixation was performed via an ilioinguinal approach with adequate reconstruction in the anterior part of the acetabulum (**D**). A postoperative CT scan shows that the posterior marginal impaction was not addressed (**E**, **F**). Thus, additional open reduction was performed via a Kocher-Langenbeck approach with surgical hip dislocation (**G**) resulting in adequate reconstruction of the articular surface (**H**). Comparison of preoperative and postoperative CT scans (**I**, **K**)

Ferguson stated that dome impactions may not be „real impactions “, but rather severe articular comminution zones [12]. In some cases posterior impactions may occur in the presence of an intact posterior column and wall. These impactions must not be ignored while performing acetabular fracture surgery (Fig. 1). Additionally, impacted fracture fragments may also prevent reduction of displaced column fracture components. Accordingly, addressing acetabular impactions intraoperatively is of utmost importance. Posterior marginal impactions are quite well understood, as they are intraoperatively visible via posterior approaches especially in posterior wall fractures and allow for open reduction under direct visualization. Surgical hip dislocation may be required occasionally. While the ilioinguinal approach was the standard anterior approach in acetabular fracture surgery for decades, several anterior approaches are available nowadays for the assessment of acetabular dome impactions, e.g. the intrapelvic approach [16–18], the Pararectus approach [11, 32] and the Smith-Petersen approach [35, 36, 41].

The aim of this review is to describe and summarize definitions, classifications, treatment options and outcomes of acetabular marginal impactions.

## Definitions

Several definitions exist for impactions in acetabular fractures:

- fragment impaction into the underlying cancellous bone from the shattered margin of the acetabulum [26].
- impacted and rotated osteocartilaginous fragment(s) of the acetabulum in conjunction with a fracture dislocation of the hip [5].
- displaced, impacted subchondral bone of the roof [1].
- dome impaction fractures, commonly independent of the anterior or posterior column fracture fragments [34].
- dome impaction with > 2 mm step-off from the original articular arc at the roof of the acetabulum [20].

In summary, there is no uniform and ubiquitously accepted definition of marginal impactions of the acetabulum.

## Classification

**Fig. 2:**
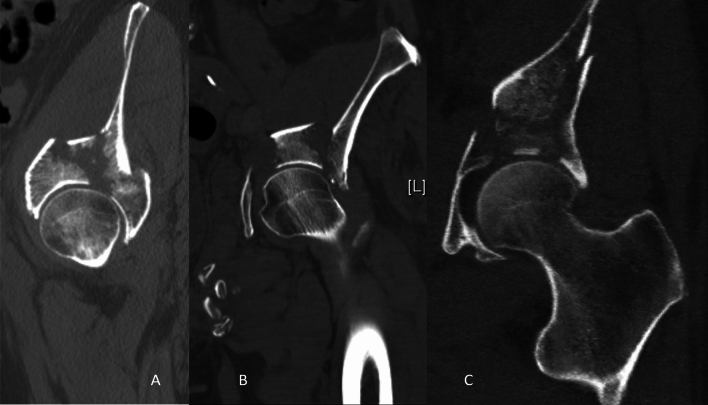
Articular steps in the anterosuperior acetabulum (**A**), intermediate fragments without impaction (**B**) and „true “ dome impactions (**C**)

**Fig. 3:**
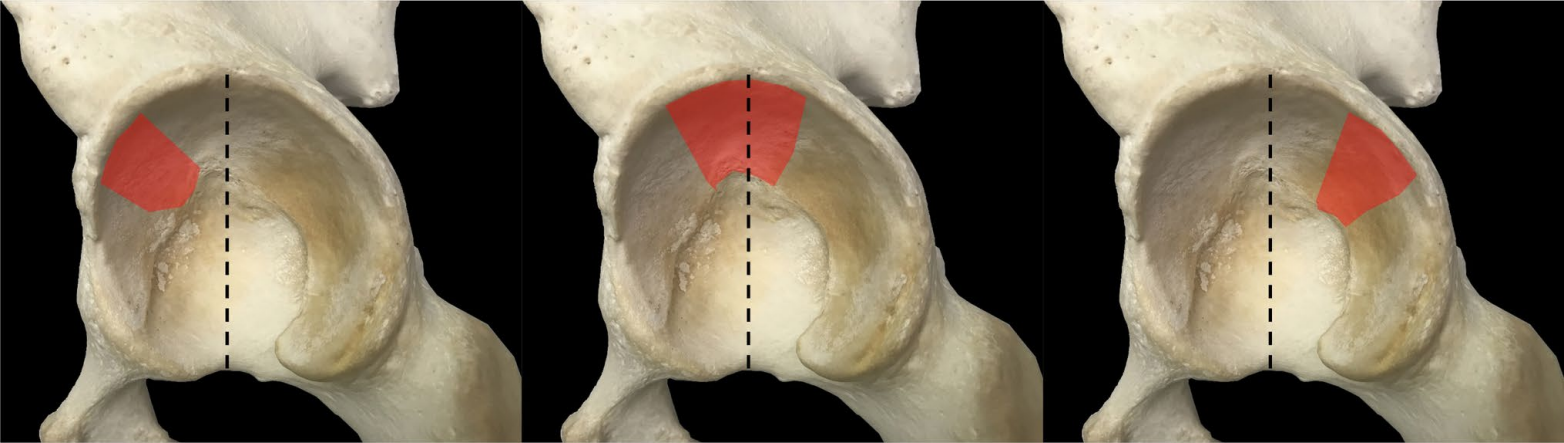
Antero-medial (left), supero-medial (middle) and postero-medial (right) acetabular dome impaction

In general, (a) posterior marginal impactions, (b) acetabular dome impactions and (c) femoral head impactions may be distinguished. Posterior marginal impactions and femoral head impactions are generally not further subclassified. This is not true for acetabular dome impactions. There are several controversities concerning acetabular dome impactions. As a first step, it is mandatory to distinguish between articular steps in the anterosuperior acetabulum, intermediate fragments without impaction and “true” dome impactions (Fig. 2; [37]). Additionally, dome impactions were described as being located anteromedially [42], superomedially [1, 7, 12, 22, 24, 43, 44] and anterosuperiorly [33]. More recently, dome impactions were subclassified into anteromedial (AM), superomedial (SM) and posteromedial (PM) dome impactions (Fig. 3) [20].

## Posterior marginal impactions

Posterior marginal impactions are common in acetabular fractures involving the posterior wall and the posterior column [14, 23, 36]. They may, however, occur without fractures of the posterior structures as well (Fig. 1). The so-called “dashboard injury” is the typical injury mechanism, which is frequently associated with posterior hip dislocation. Depending on the amount of hip flexion during trauma, the impactions may have a pure posterior or a more posterosuperior or -inferior location, respectively.

**Fig. 4:**
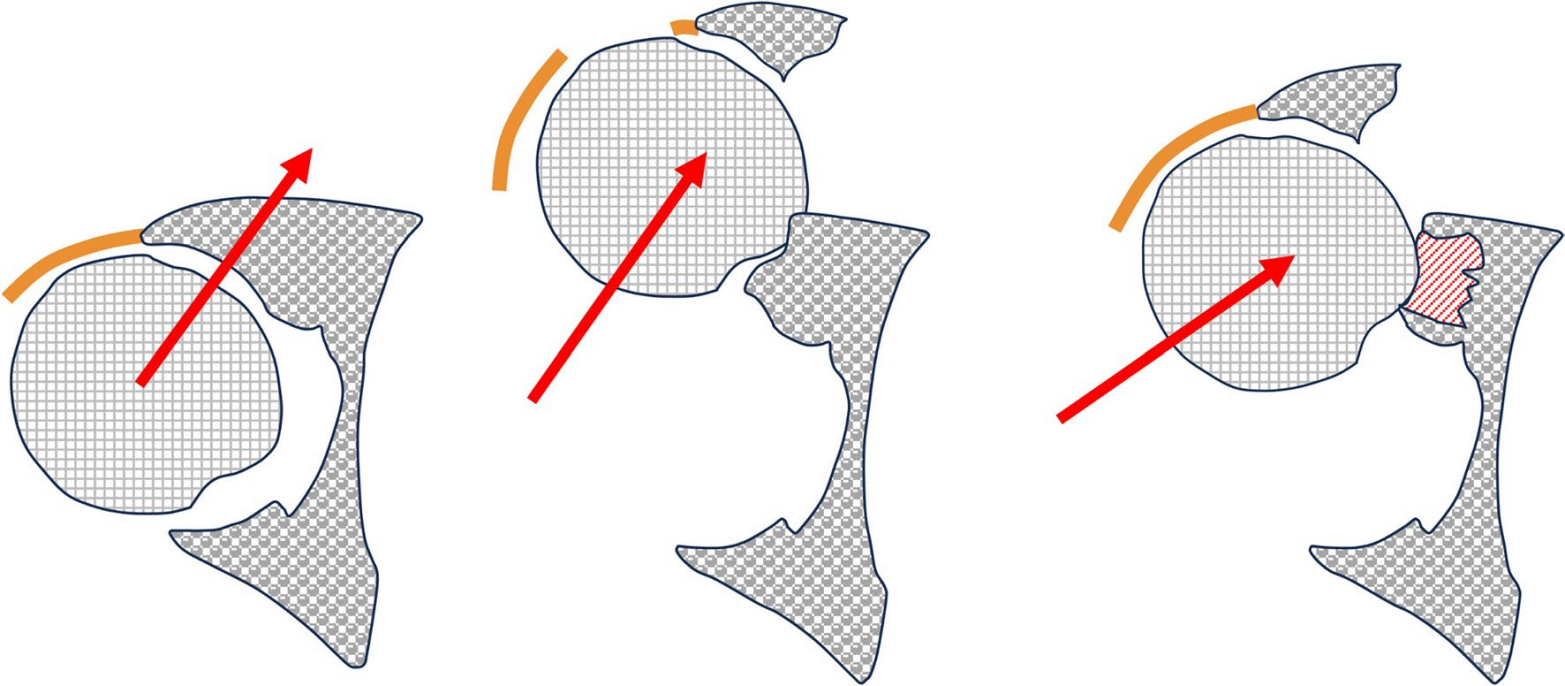
Injury mechanism of posterior marginal impactions as described by Letournel (left). Associated capsular disruption predominantly leads to isolated fragments (middle), while a remaining intact capsule attached to the posterior wall fragment is associated with a higher risk of marginal impaction (right)

**Fig. 5:**
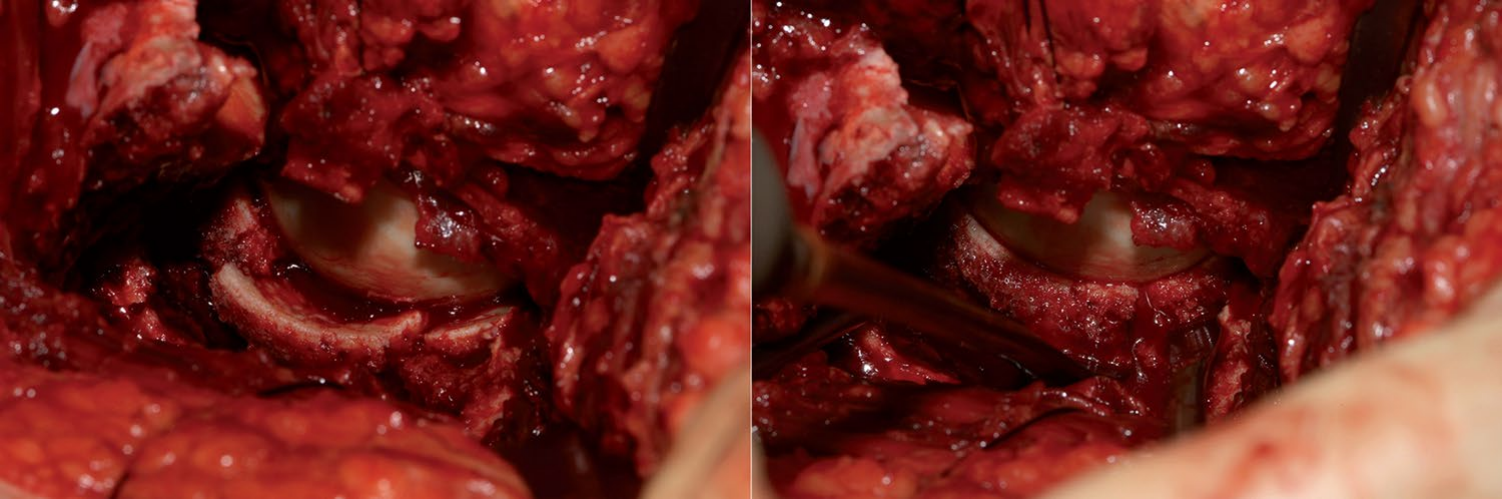
Multiple posterior impactions in an associated posterior column and posterior wall fracture before (left) and after open reduction (right)

A posterior wall fragment may either remains attached to the joint capsule or an additional rupture of the capsule may occur (Fig. 4). In the presence of an intact capsule the femoral head is directed to the remaining posterior osseous structures by ligamentotaxis and may cause marginal impaction there (Figs. 4 and 5). These injury mechanism also frequently result in damage of the femoral head cartilage, in femoral head impactions or even in femoral head fractures (Pipkin type IV).

Brumback reported on a series of 17 patients with posterior fracture dislocations with associated impactions [5]. They found a rate of 23% of posterior marginal impactions in posterior hip dislocations. Giannoudis reported on acetabular fractures with impaction in a large series of 320 patients [13]. It was noted that reduction of the hip dislocation does not result in reduction of the impaction. This is an obvious finding, as there is no soft-tissue attachment on the impacted fragments and therefore direct open reduction is mandatory. The authors found that posterior impactions were more frequently than dome impactions, while femoral head impactions were found in 15% of the cases. The prevalence of posterior marginal impaction was 52% in elementary posterior wall fractures and 52.6% in associated posterior wall fractures (posterior column and posterior wall fractures, transverse and posterior wall fractures). In T-type fractures posterior marginal impactions were observed in 13.3% of the cases.

Perumal reported on the results of 16 patients with posterior marginal impactions treated surgically via posterior approaches [29]. Surgery consisted of open reduction, filling of the void with allograft bone and internal fixation. They described a rate of anatomical reduction of 75%, while the reduction was imperfect in 25% of the cases. The clinical results were graded as good or excellent in 62.5% of the cases. The authors did not observe loss of reduction in any case.

## Acetabular dome impactions

The typical injury mechanism resulting in acetabular dome impactions is a fall to the lateral side with the hip joint in extension. This results in force transmission from lateral to medial via the greater trochanter and the femoral neck. Depending on the amount of hip rotation during trauma, the impactions may be located anteromedially, superomedially or posteromedially, respectively. This is a frequent injury mechanism in the elderly [34]. Accordingly, the relative frequency of acetabular dome impactions is positively correlated with the patients´ age, while this is not true for posterior marginal impactions.

The “gull sign” [1] was described as the 2D fluoroscopic appearance of acetabular dome impactions. Tosounidis stated that the gull sign corresponds to an anteromedial dome impaction [42], while other authors correspond the gull sign to a superomedial dome impaction [6, 7, 44]. In a recent study it was shown that superomedial impactions in a CT scan corresponded to a gull sign in more than 90% of the cases, while the rate was 40% for posteromedial impactions and 25% only for anteromedial impactions [20]. Accordingly, dome impactions may not be assessable in X-rays (ap view), if they are mainly located anteriorly (Fig. 3, left) or posteriorly (Fig. 3, right). A CT scan with thin slices is therefore mandatory for the assessment of dome impactions.

The reduction of acetabular dome impactions is of utmost importance for the long-term functional outcome and to restore hip containment. In contrast to posterior impactions, however, this may be very difficult due to the restricted access to the joint provided by anterior approaches. Mainly two reduction techniques were proposed in the literature, namely reduction via a cortical window and reduction via fracture lines if possible.

**Fig. 6:**
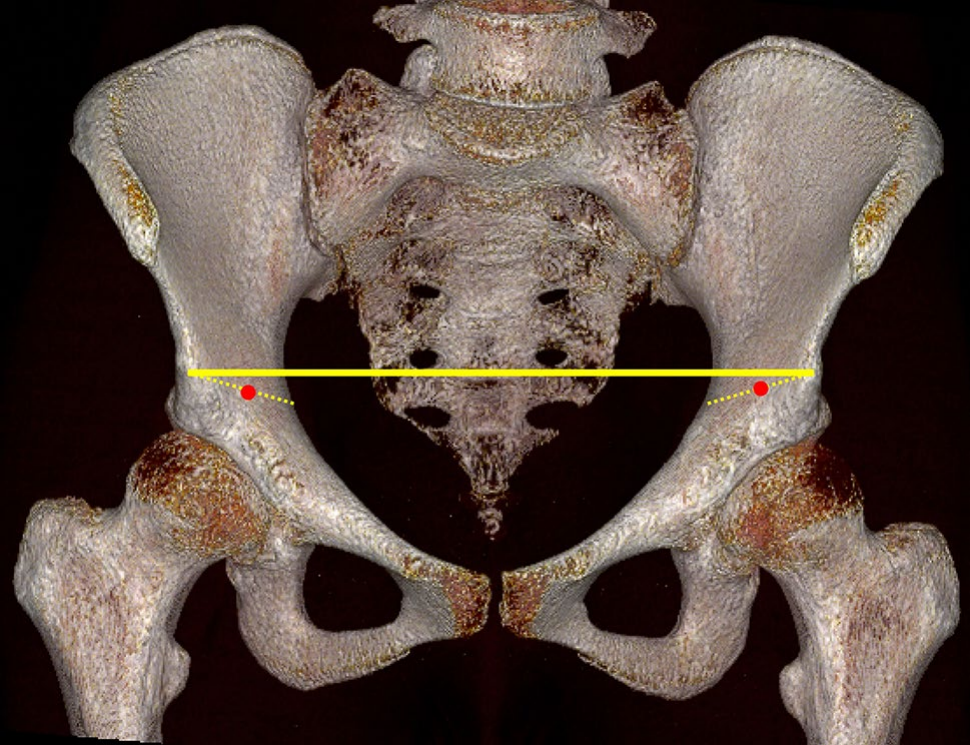
Identification of the area of a cortical window for reduction of superomedial dome impactions: the midpoint (red dot) of the reflection (dotted yellow line) of a line between both anterior inferior iliac spines (yellow line) provides a good approximation of the location for a cortical window

Reduction of articular impactions via a cortical window is a well known technique mainly from pure depression fractures of the lateral tibial head. A ball-spike pusher can be used for reduction followed by filling the void with cancellous bone, allogenic bone or bone substitutes supported by raft screws [34, 42]. The cortical window technique is mainly useful for superomedial and some posteromedial dome impactions [20, 21]. The location of the required cortical window was recently defined in a cadaver- and CT-based study [19]. An intraoperative reference area was defined in the anterior and superior direction of the impaction area. To identify this area, a line connecting both AIIS is reflected to the supraacetabular bone surface. The midpoint of this reflected line marks the area for the cortical window (Fig. 6).

**Fig. 7:**
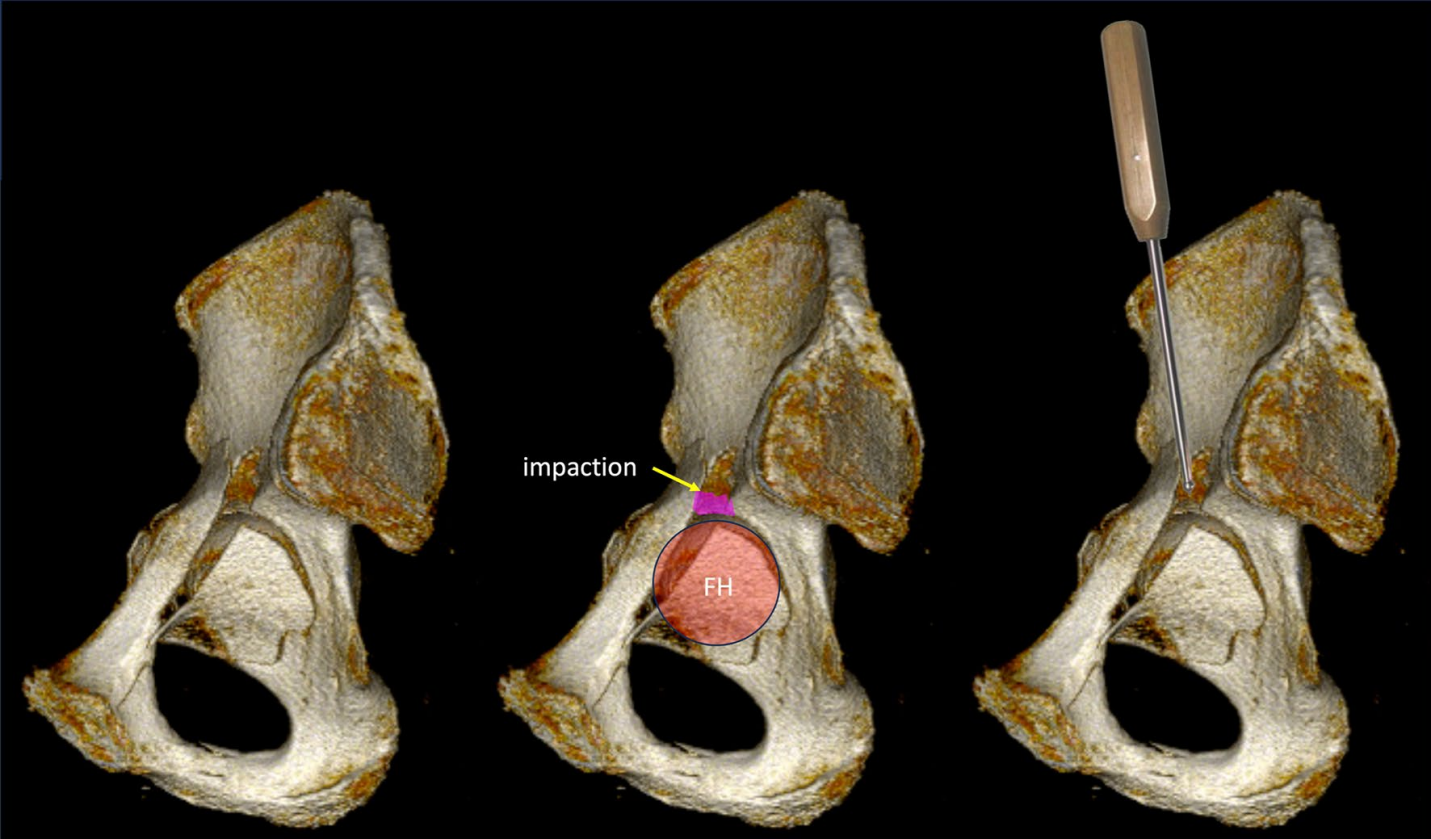
Reduction of a superomedial impaction with a ball-spike pusher through a fracture line using the femoral head as a template

Reduction via fracture lines is mainly used in anteromedial and posteromedial impactions, but may be advisable in superomedial dome impactions as well (Fig. 7). For anteromedial impactions, reduction can be performed through the fracture line of the anterior column between the pubic ramus and the iliac fragment prior to reduction of the anterior column [45]. For posteromedial impactions, a fracture line between the anterior and the posterior column, which is usually located below the pelvic brim, may be used.

In general, reduction of the acetabular columns may be performed prior to the dome disimpaction, when applying the cortical window technique, or after column reduction, when using fracture lines for the reduction of the impactions [7, 24]. It is highly recommended to reduce the femoral head by lateral traction first. The femoral head then serves as a template for disimpaction (Fig. 7). Medial buttress plating is the gold standard for definitive fixation after reduction of dome impactions to address the medial wall and acetabular column fractures [7, 8, 24, 42].

**Fig. 8:**
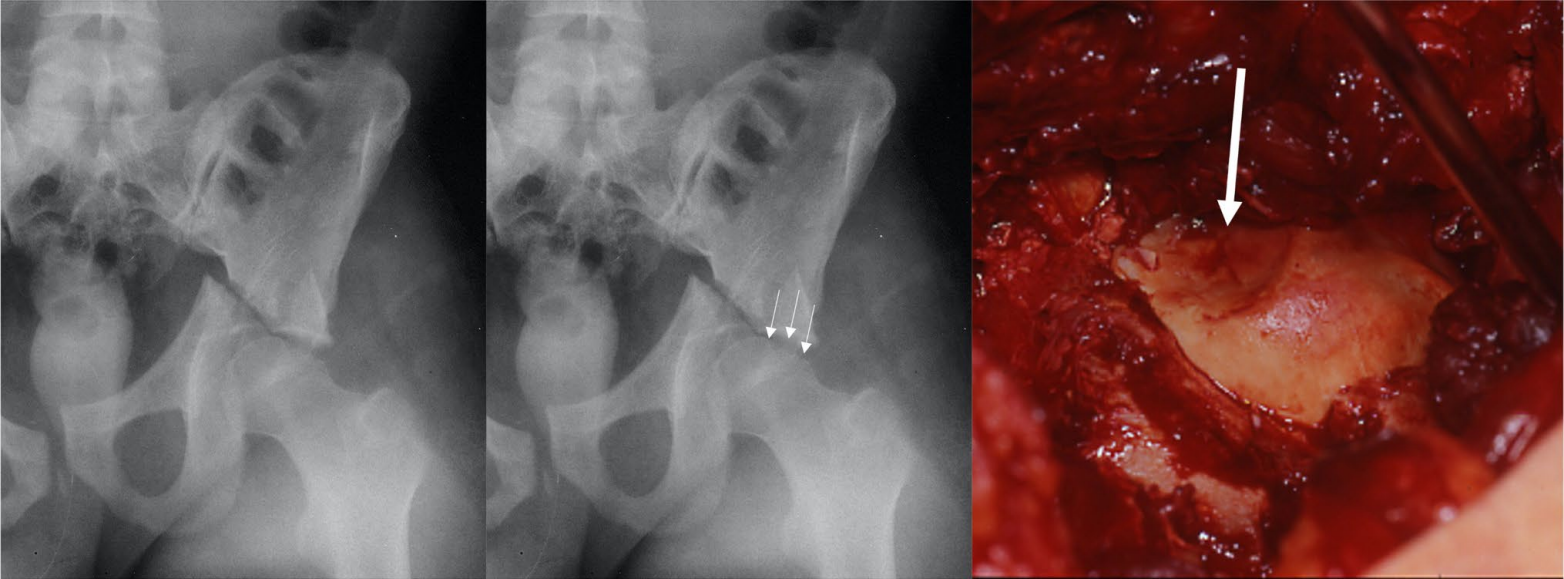
Superior femoral head impaction (arrows) in a patient with a transverse acetabular fracture. The femoral head is typically impacted by the medial edge of the iliac fragment

Laflamme described a detailed technique for the reduction of superomedial dome impactions. Reduction is performed either using the anterior intrapelvic approach with and additional lateral iliac window or the ilioinguinal approach with a medial intrapelvic window [24]. After reconstruction of the iliac wing using lateral femoral head traction, the intrapelvic window is used for tilting the quadrilateral plate fragment medially and posteroinferiorly. This allows for a direct assessment of the superomedial dome impaction. Reduction is then performed with a periosteal elevator using the reduced femoral head as a template under fluoroscopic control (AP view and obturator oblique view). The void is preferably filled with calcium phosphate, as it is stronger than bone grafts. However, autologous bone from the iliac crest or the greater trochanter is a valuable alternative option. Raft screw fixation is then performed from lateral to medialor vice versa. This is followed by quadrilateral plate fixation. The authors reported on 9 patients with superomedial impactions using the descibed technique. There were two cases with malreduction and one case with loss of correction due to suboptimal raft screw positioning [24] (Fig. 8).

## Femoral head impactions

Letournel stated that „it is not surprising that there is a considerable incidence of damage to the femoral head in view of the magnitude of the force necessary to produce a fracture of the acetabulum and the fact that it is the femoral head which transmits this force” [27]. Femoral head damage may occur as (a) femoral head fractures (aka Pipkin fractures), as (b) femoral head impactions or as (c) damage of the cartilage without osseous damage. Femoral head impactions will be discussed in this chapter only. They are relevant risk factor for posttraumatic osteoarthritis following acetabular fractures with an odds ratio of 4.8 [32].

On the one hand, femoral head impactions occur in hip dislocations as a result of shearing forces between the femoral head and the acetabular rim. These injuries therefore correspond to Hill–Sachs lesions in glenohumeral dislocations [31]. On the other hand, they occur following an impact of the femoral head with the acetabular cavity or fracture fragments. No dislocation is observed in theses cases.

Historical data reported rates of femoral head impaction following hip dislocation of 7–16% [10, 25, 28, 30]. Letournel observed superolateral impactions (5–15 mm) of the femoral head in 2.1% of the cases of hip dislocation only. The problem with these historical data is the fact that femoral head impactions are frequently not diagnosed on conventional X-rays [38]. They may be even hard to detect in CT scans with low resolution or impaired quality. In a CT study from 1990, a rate of 62.5% of anterior femoral head impactions was found in posterior hip dislocations. Vice versa, posterior impactions were found in anterior hip dislocations [40]. Ferguson found a relative frequency of 19.6% in 173 patients aged > 60 years. This relatively low rate may be attributed to the minor force impact occurring in the elderly [12]. In a recent study from 2019 including 128 acetabular fracture patients, femoral head impactions were found in 3.9% of the patients only by the radiologists in the initial reading. Intraoperatively an additional rate of 2.3% were found. Reevaluations of the initial CT scans showed a rate of 39.8%. Accordingly, it is reasonable to assume that femoral head impactions remain frequently undiagnosed. The authors found that anterosuperior impactions were mainly found in patients without hip dislocation, while hip dislocation more frequently resulted in anteroinferior impactions. Superolateral impactions were frequently observed in patients with transverse acetabular fractures.

Some case reports reporting on the treatment of deep femoral head impactions using osteochondral grafts [2, 3, 9] are reported in the literature and summarized by Hanke [15]. Surgical hip dislocation was mainly chosen as an approach with the direct anterior approach serving as an alternative option. Accordingly, these are the same approaches, which are used for the surgical treatment of Pipkin fractures. There is a paucity of data regarding the long-term outcome of these procedures. Hanke reported on a series of 12 patients with a minimum follow-up of 5 years. The rate of hip survival was 57% [15].

In conclusion, there is no gold standard for the treatment of deep femoral head impactions due to the lack of data on their long term outcome. Superficial femoral head impactions are typically not addressed surgically.

## Data Availability

No datasets were generated or analysed during the current study.
